# Crystal structures of three co-crystals of 4,4′-bipyridyl with 4-alk­oxy­benzoic acids: 4-eth­oxy­benzoic acid–4,4′-bipyridyl (2/1), 4-*n*-propoxybenzoic acid–4,4′-bipyridyl (2/1) and 4-*n*-but­oxy­benzoic acid–4,4′-bipyridyl (2/1)

**DOI:** 10.1107/S2056989015018435

**Published:** 2015-10-07

**Authors:** Yohei Tabuchi, Kazuma Gotoh, Hiroyuki Ishida

**Affiliations:** a Department of Chemistry, Faculty of Science, Okayama University, Okayama 700-8530, Japan

**Keywords:** crystal structure, 4,4′-bipyrid­yl, 4-alk­oxy­benzoic acid, hydrogen-bonded liquid crystal

## Abstract

The crystal structures of three hydrogen-bonded co-crystals of 4-alk­oxy­benzoic acid–4,4′-bipyridyl (2/1), namely, 2C_9_H_10_O_3_·C_10_H_8_N_2_, (I), 2C_10_H_12_O_3_·C_10_H_8_N_2_, (II) and 2C_11_H_14_O_3_·C_10_H_8_N_2_, (III), have been determined at 93 K. Although the structure of (I) has been determined in the space group *P*2_1_ with *Z* = 4 [Lai *et al.* (2008 ▸). *J. Struct. Chem.* **49**, 1137–1140], the present study shows that the space group is *P*2_1_/*n* with *Z* = 4. In each crystal, the components are linked by O—H⋯N hydrogen bonds, forming a linear hydrogen-bonded 2:1 unit of the acid and the base. The 2:1 unit of (I) adopts nearly pseudo-*C*_2_ symmetry, *viz*. twofold rotation around an axis passing through the mid-point of the central C—C bond of 4,4′-bipyridyl, while the units of (II) and (III), except for the terminal alkyl chains, have pseudo-inversion symmetry. The 2:1 units of (I), (II) and (III) are linked *via* C—H⋯O hydrogen bonds, forming sheet, double-tape and tape structures, respectively.

## Abstract 2

Crystal structures of three co-crystals of bis­(4-alk­oxy­benzoic acid) and 4,4′-bipyridyl have been determined at 93 K. The asymmetric unit of each compound comprises two crystallographically independent acid mol­ecules and one base mol­ecule, which are held together by O—H⋯N hydrogen bonds, forming a linear hydrogen-bonded 2:1 unit.

## Chemical context   

The 4-alk­oxy­benzoic acid–4,4′-bipyridyl (2/1) system, in which the two acids and the base are held together by inter­molecular O—H⋯N hydrogen bonds, shows thermotropic liquid crystallinity (Kato *et al.*, 1990 ▸, 1993 ▸; Grunert *et al.*, 1997 ▸). The compounds of 4-meth­oxy-, 4-eth­oxy- and 4-*n*-propoxybenzoic acid show nematic phases, while the compound of 4-*n*-but­oxy­benzoic acid exhibits a smectic A phase and then a nematic phase with increasing temperature (Kato *et al.*, 1990 ▸, 1993 ▸). The crystal structure of 4-meth­oxy­benzoic acid–4,4′-bipyridyl (2/1) was reported recently (Mukherjee & Desiraju, 2014 ▸; Ramon *et al.*, 2014 ▸). Although the structure of 4-eth­oxy­benzoic acid–4,4′-bipyridyl (2/1) in space group *P*2_1_ was also reported (Lai *et al.*, 2008 ▸), the mol­ecular structure is distorted probably due to the wrong choice of space group. In the present study, we have analysed the structure of 4-eth­oxy­benzoic acid–4,4′-bipyridyl (2/1), (I), as well as the structures of 4-*n*-propoxybenzoic acid–4,4′-bipyridyl (2/1), (II), and 4-*n*-but­oxy­benzoic acid–4,4′-bipyrid­yl(2/1), (III).
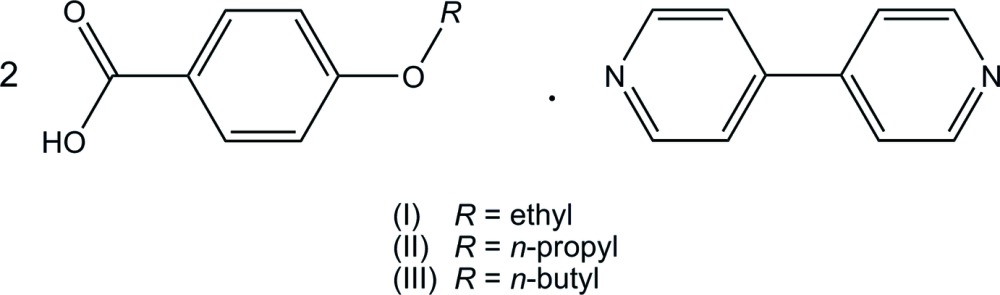


## Structural commentary   

**Figure 1:**
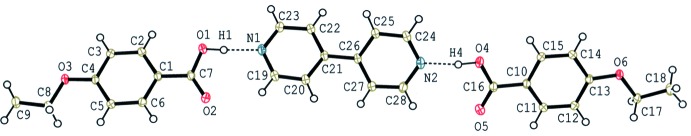
The mol­ecular structure of compound (I), showing the atom-numbering scheme. Displacement ellipsoids of non-H atoms are drawn at the 50% probability level and H atoms are drawn as circles of arbitrary size. The O—H⋯N hydrogen bonds are indicated by dashed lines.

**Figure 2:**
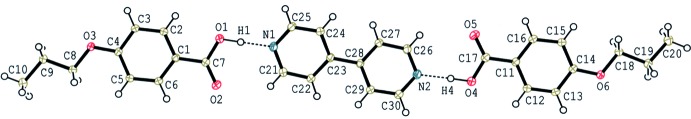
The mol­ecular structure of compound (II), showing the atom-numbering scheme. Displacement ellipsoids of non-H atoms are drawn at the 50% probability level and H atoms are drawn as circles of arbitrary size. The O—H⋯N hydrogen bonds are indicated by dashed lines.

The mol­ecular structure of (I) is shown in Fig. 1 ▸. Compound (I) crystallizes in the space group *P*2_1_/*n* with *Z* = 4. For the structure (space group *P*2_1_) previously determined by Lai *et al.* (2008 ▸), *ADDSYM* in *PLATON* (Spek, 2009 ▸) detected missed symmetry elements, *viz.* a centre of inversion and a glide plane. The mol­ecular structures of (II) and (III) are shown in Figs. 2 ▸ and 3 ▸, respectively. The asymmetric units each comprise two crystallographically independent 4-alk­oxy­benzoic acid mol­ecules and one 4,4′-bipyridyl mol­ecule, and the two acids and the base are held together by O—H⋯N hydrogen bonds (Tables 1 ▸, 2 ▸ and 3 ▸), forming a linear hydrogen-bonded 2:1 aggregate. Similar to the reported structure of the 2:1 unit of 4-meth­oxy­benzoic acid–4,4′-bipyridyl (2/1) (Mukherjee & Desiraju, 2014 ▸; Ramon *et al.*, 2014 ▸), the 2:1 unit of (I) also adopts nearly pseudo-*C*_2_ symmetry, *viz.* twofold rotation around an axis passing through the mid-point of the central C21—C26 bond of the 4,4′-bipyridyl mol­ecule. On the other hand, the 2:1 units of (II) and (III), except for the terminal alkyl chains, have pseudo-inversion symmetry.

The dihedral angles between the pyridine rings of 4,4′-bipyridyl are 27.95 (5), 28.84 (4) and 38.76 (12)° for (I), (II) and (III), respectively. The pyridine ring and the carboxyl group hydrogen-bonded to it are twisted slightly to each other. The dihedral angles between the N1/C19–C23 and O1/O2/C7 planes, and the N2/C24–C28 and O4/O5/C16 planes are 6.54 (11) and 10.31 (11)°, respectively, in (I), those between the N1/C21–C25 and O1/O2/C7 planes, and the N2/C26–C30 and O4/O5/C17 planes are 12.13 (10) and 13.96 (10)°, respectively, in (II), and those between the N1/C23–C27 and O1/O2/C7 planes, and the N2/C28–C32 and O4/O5/C18 planes are 13.7 (3) and 8.5 (3)°, respectively, in (III).

The mol­ecular structures of the eth­oxy- and propoxy­benzoic acids in (I) and (II) are approximately planar. The dihedral angles made by the benzene ring with the carboxyl group and the alk­oxy group in each eth­oxy­benzoic acid in (I) are 9.60 (10), 1.13 (11), 4.48 (9) and 7.57 (9)°, respectively, between the C1–C6 and O1/O2/C7 planes, the C10–C15 and O4/O5/C16 planes, the C1–C6 and O3/C8/C9 planes, and the C10–C15 and O6/C17/C18 planes. The corresponding dihedral angles in (II) are 2.42 (10), 2.48 (10), 2.96 (7) and 5.82 (7)°, respectively, between the C1–C6 and O1/O2/C7 planes, the C11–C16 and O4/O5/C17 planes, the C1–C6 and O3/C8/C9/C10 planes, and the C11–C16 and O6/C18/C19/C20 planes. The but­oxy­benzoic acid mol­ecules in (III) are also planar, except for the terminal ethyl groups which deviate from the mol­ecular plane with dihedral angles of 66.6 (3) and 60.7 (3)°, respectively, between the C4/O3/C8 and C9/C10/C11planes, and the C15/O6/C19 and C20/C21/C22 planes. The dihedral angles made by the benzene ring with the carboxyl group and the alk­oxy group are 5.6 (3), 5.4 (3), 5.2 (2) and 4.3 (2)°, respectively, between the C1–C6 and O1/O2/C7 planes, the C12–C17 and O4/O5/C18 planes, the C1–C6 and O3/C8/C9 planes, and the C11–C16 and O6/C19/C20 planes.

## Supra­molecular features   

**Figure 4:**
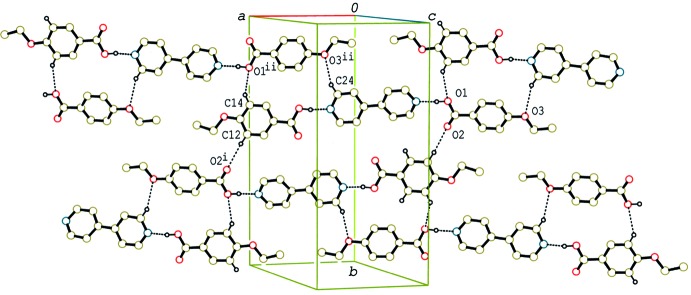
A partial packing diagram of compound (I), showing the sheet structure formed by O—H⋯N and C—H⋯O hydrogen bonds (dashed lines). H atoms not involved in the hydrogen bonds have been omitted. [Symmetry codes: (i) −*x* + 1, −*y* + 1, −*z* + 1; (ii) *x* + 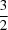, −*y* + 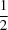, *z* − 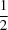.]

**Figure 5:**
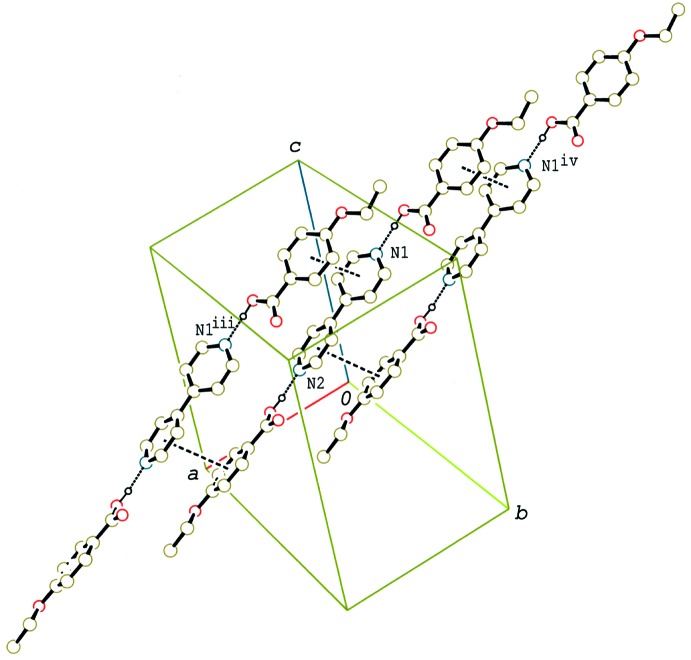
A partial packing diagram of compound (I), showing the column structure formed by π–π stacking inter­actions (dashed lines). H atoms not involved in the O—H⋯N hydrogen bonds have been omitted. [Symmetry codes: (iii) *x* + 1, *y*, *z*; (iv) *x* − 1, *y*, *z*.]

In the crystal of (I), the 2:1 units are linked by C—H⋯O hydrogen bonds (Table 1 ▸), forming a sheet structure parallel to (103) (Fig. 4 ▸). In addition, the units are stacked in a column through π–π inter­actions between the acid and base rings along the *a* axis (Fig. 5 ▸). The centroid–centroid distances between the C1–C6 and N1/C19–C23(*x* − 1, *y*, *z*) rings, and between the C10–C15 and N2/C24–C28 (*x* + 1, *y*, *z*) rings are 3.7052 (5) and 3.7752 (6) Å, respectively. C—H⋯π inter­actions (Table 1 ▸) are also observed between the columns and between the sheets.

**Figure 6:**
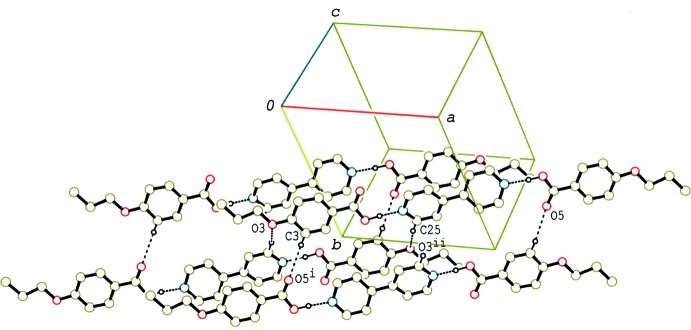
A partial packing diagram of compound (II), showing the double-tape structure formed by C—H⋯O inter­actions. H atoms not involved in the C—H⋯O and O—H⋯N hydrogen bonds (dashed lines) have been omitted. [Symmetry codes: (i) −*x* + 1, −*y* + 2, −*z*; (ii) −*x*, −*y* + 2, −*z*.]

**Figure 7:**
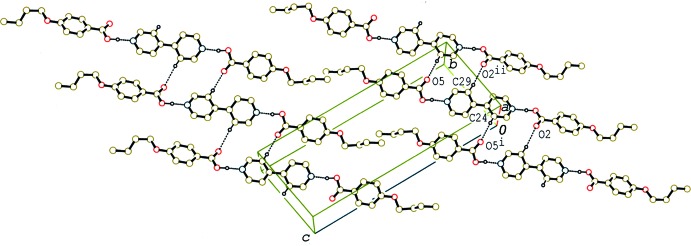
A partial packing diagram of compound (III), showing the tape structure formed by C—H⋯O inter­actions. H atoms not involved in the C—H⋯O and O—H⋯N hydrogen bonds (dashed lines) have been omitted. [Symmetry codes: (i) *x*, *y* − 1, *z*; (ii) *x*, *y* + 1, *z*.]

**Figure 8:**
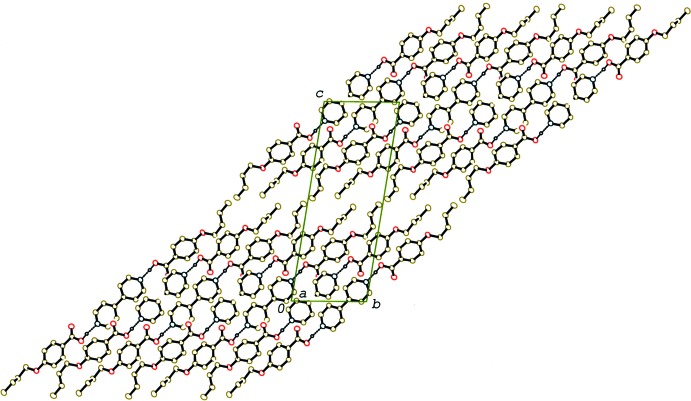
A packing diagram of compound (III) viewed along the *a* axis, showing a layer aggregate. H atoms not involved in the O—H⋯N hydrogen bonds (dashed lines) have been omitted.

In the crystal of (II) and (III), the 2:1 units are linked by C—H⋯O inter­actions (Tables 2 ▸ and 3 ▸), forming a double-tape structure along the *a* axis (Fig. 6 ▸) and a tape structure along the *b* axis (Fig. 7 ▸), respectively. Between the tapes in (II) and (III) C—H⋯π inter­actions are observed (Tables 2 ▸ and 3 ▸). A packing diagram of (III) viewed along the *a* axis, which is approximately perpendicular to the mean plane of the 2:1 unit, is shown in Fig. 8 ▸. The units are arranged into a layer parallel to the *bc* plane, which leads to a smectic structure. On the other hand, no such a layer structure is observed in compounds (I) and (II), which form nematic liquid phases.

## Database survey   

A search of the Cambridge Structural Database (Version 5.36, last update February 2015; Groom & Allen, 2014 ▸) for co-crystals of 4,4′-bipyridyl with 4-alk­oxy­benzoic acid gave five structures (refcodes: NOPXIZ, ORASAC, RIRGUV, YAKVAI and YANCUM), except for 4-meth­oxy­benzoic acid–4,4′-bipyridyl (2/1) and 4-eth­oxy­benzoic acid–4,4′-bipyridyl (2/1). Of these compounds, NOPXIZ, 4-[(*S*)-(−)-2-methyl­but­oxy]benzoic acid–4,4′-bipyridyl (2/10, shows smectic A and nematic phases (Grunert *et al.*, 1997 ▸).

## Synthesis and crystallization   

Single crystals of compound (I) were obtained by slow evaporation from an acetone solution (150 ml) of 4,4′-bipyridyl (70 mg) with 4-eth­oxy­benzoic acid (150 mg) at room temperature. Crystals of compounds (II) and (III) were obtained from ethanol solutions of 4,4′-bipyridyl with 4-*n*-propoxybenzoic acid and 4-*n*-but­oxy­benzoic acid, respectively, at room temperature [ethanol solution (150 ml) of 4,4′-bipyridyl (65 mg) and 4-*n*-propoxybenzoic acid (150 mg) for (II), and ethanol solution (150 ml) of 4,4′-bipyridyl (60 mg) and 4-*n*-but­oxy­benzoic acid (150 mg) for (III)].

Liquid crystalline phases of these compounds were confirmed by measurements of DSC (differential scanning calorimetry) and polarizing microscope. DSC measurements were performed by using Perkin Elmer Pyris 1 in the temperature range from 103 K to the melting temperature at a heating rate of 10 K min^−1^. Phase transition temperatures (K) and enthalpies (kJ mol^−1^) determined by DSC are as follows:

(I) 373 (2) [5.4 (4)] K_1_ → K_2_, 424 (1) [50 (3)] K_2_ → N, 442 (1) [7.2 (6)] N → I;

(II) 365 (1) [2.9 (6)] K_1_ → K_2_, 369 (1) [3.9 (2)] K_2_ → K_3_, 417 (1) [39 (1)] K_3_ → N, 430 (1) [5.7 (2)] N → I;

(III) 358 (1) [2.5 (2)] K_1_ → K_2_, 386 (1) [0.30 (3)] K_2_ → K_3_, 403 (1) [11.1 (5)] K_3_ → K_4_, 407 (1) [24.5 (6)] K_4_ → S_A_, 425 (1) [2.2 (6)] S_A_ → N, 432 (1) [6.4 (1)] N → I.

K_i_, S_A_, N and I denote crystal, smectic A, nematic and isotropic phases, respectively. The observed transition temperatures and enthalpies from the solid phase to the liquid crystalline phase are in good agreement with those reported Kato *et al.* (1990 ▸, 1993 ▸). Some unreported thermal anomalies, 373 (2) K for (I), 365 (1) and 369 (1) K for (II), and 358 (1) and 386 (1) K for (III), were also observed.

## Refinement   

Crystal data, data collection and structure refinement details are summarized in Table 4 ▸. For all compounds, C-bound H atoms were positioned geometrically with C—H = 0.95–0.99 Å and were refined as riding with *U*_iso_(H) = 1.2*U*_eq_(C) or 1.5*U*_eq_(methyl C). The O-bound H atoms were located in a difference Fourier map and refined freely [refined O—H = 0.942 (19)–1.04 (3) Å].

## Supplementary Material

Crystal structure: contains datablock(s) I, II, III, General. DOI: 10.1107/S2056989015018435/lh5794sup1.cif

Structure factors: contains datablock(s) I. DOI: 10.1107/S2056989015018435/lh5794Isup2.hkl

Structure factors: contains datablock(s) II. DOI: 10.1107/S2056989015018435/lh5794IIsup3.hkl

Structure factors: contains datablock(s) III. DOI: 10.1107/S2056989015018435/lh5794IIIsup4.hkl

CCDC references: 1429203, 1429204, 1429205

Additional supporting information:  crystallographic information; 3D view; checkCIF report

## Figures and Tables

**Figure 3:**
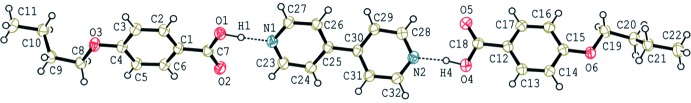
The mol­ecular structure of compound (III), showing the atom-numbering scheme. Displacement ellipsoids of non-H atoms are drawn at the 50% probability level and H atoms are drawn as circles of arbitrary size. The O—H⋯N hydrogen bonds are indicated by dashed lines.

**Table 1:** Hydrogen-bond geometry (Å, °) for (I) *Cg*1 and *Cg*2 are the centroids of the C1–C6 and C10–C15 rings, respectively.

*D*—H⋯*A*	*D*—H	H⋯*A*	*D*⋯*A*	*D*—H⋯*A*
O1—H1⋯N1	0.942 (19)	1.72 (2)	2.6587 (11)	177.2 (19)
O4—H4⋯N2	0.948 (19)	1.690 (19)	2.6312 (11)	171.4 (19)
C12—H12⋯O2^i^	0.95	2.43	3.3712 (11)	172
C14—H14⋯O1^ii^	0.95	2.56	3.2288 (11)	128
C24—H24⋯O3^ii^	0.95	2.57	3.4407 (12)	153
C9—H9*A*⋯*Cg*2^iii^	0.98	2.68	3.6450 (11)	169
C18—H18*C*⋯*Cg*1^iv^	0.98	2.67	3.6253 (11)	164

Symmetry codes: (i) 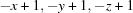; (ii) 
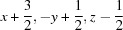
; (iii) 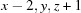; (iv) 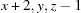.

**Table 2:** Hydrogen-bond geometry (Å, °) for (II) *Cg*1 and *Cg*2 are the centroids of the C1–C6 and C11–C16 rings, respectively.

*D*—H⋯*A*	*D*—H	H⋯*A*	*D*⋯*A*	*D*—H⋯*A*
O1—H1⋯N1	1.03 (2)	1.61 (2)	2.6407 (10)	174.3 (19)
O4—H4⋯N2	1.01 (2)	1.67 (2)	2.6728 (11)	173.9 (18)
C3—H3⋯O5^i^	0.95	2.57	3.3981 (11)	146
C25—H25⋯O3^ii^	0.95	2.57	3.4581 (11)	156
C9—H9*B*⋯*Cg*2^iii^	0.99	2.84	3.6750 (1)	142
C19—H19*A*⋯*Cg*1^iv^	0.99	2.72	3.5781 (1)	146

Symmetry codes: (i) 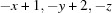; (ii) 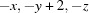; (iii) 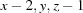; (iv) 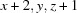.

**Table 3:** Hydrogen-bond geometry (Å, °) for (III) *Cg*1 and *Cg*2 are the centroids of the C1–C6 and C12–C17 rings, respectively.

*D*—H⋯*A*	*D*—H	H⋯*A*	*D*⋯*A*	*D*—H⋯*A*
O1—H1⋯N1	1.04 (3)	1.56 (3)	2.600 (3)	173 (3)
O4—H4⋯N2	1.00 (4)	1.64 (4)	2.636 (3)	172 (4)
C24—H24⋯O5^i^	0.95	2.47	3.408 (3)	171
C29—H29⋯O2^ii^	0.95	2.53	3.456 (3)	164
C2—H2⋯*Cg*2^iii^	0.95	2.98	3.754 (3)	139
C8—H8*B*⋯*Cg*2^iv^	0.99	2.68	3.518 (3)	143
C19—H19*B*⋯*Cg*1^v^	0.99	2.77	3.586 (3)	140

Symmetry codes: (i) 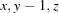; (ii) 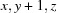; (iii) 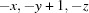; (iv) 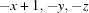; (v) 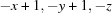.

**Table 4:** Experimental details

	(I)	(II)	(III)
Crystal data			
Chemical formula	2C_9_H_10_O_3_·C_10_H_8_N_2_	2C_10_H_12_O_3_·C_10_H_8_N_2_	2C_11_H_14_O_3_·C_10_H_8_N_2_
*M*_r_	488.52	516.57	544.63
Crystal system, space group	Monoclinic, *P*2_1_/*n*	Triclinic, *P* 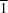	Triclinic, *P* 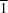
Temperature (K)	93	93	93
*a*, *b*, *c* (Å)	9.1090 (2), 20.9348 (5), 12.8738 (4)	10.7592 (4), 10.8838 (3), 11.6462 (4)	7.6645 (10), 8.5087 (13), 22.606 (3)
α, β, γ (°)	90, 102.9429 (10), 90	86.6411 (11), 89.2313 (13), 73.8867 (12)	80.498 (3), 86.486 (3), 80.082 (3)
*V* (Å^3^)	2392.60 (11)	1307.95 (8)	1431.5 (4)
*Z*	4	2	2
Radiation type	Mo *K*α	Mo *K*α	Mo *K*α
μ (mm^−1^)	0.10	0.09	0.09
Crystal size (mm)	0.28 × 0.25 × 0.10	0.50 × 0.40 × 0.10	0.53 × 0.41 × 0.11

Data collection			
Diffractometer	Rigaku R-AXIS RAPIDII	Rigaku R-AXIS RAPIDII	Rigaku R-AXIS RAPIDIIr
No. of measured, independent and observed [*I* > 2σ(*I*)] reflections	28629, 6941, 6004	15909, 7507, 5980	12433, 5612, 3432
*R*_int_	0.035	0.069	0.075
(sin θ/λ)_max_ (Å^−1^)	0.703	0.703	0.617

Refinement			
*R*[*F* ^2^ > 2σ(*F* ^2^)], *wR*(*F* ^2^), *S*	0.045, 0.127, 1.04	0.047, 0.135, 1.04	0.069, 0.193, 1.01
No. of reflections	6941	7507	5610
No. of parameters	335	354	371
H-atom treatment	H atoms treated by a mixture of independent and constrained refinement	H atoms treated by a mixture of independent and constrained refinement	H atoms treated by a mixture of independent and constrained refinement
Δρ_max_, Δρ_min_ (e Å^−3^)	0.39, −0.37	0.35, −0.32	0.24, −0.41

Computer programs: *RAPID-AUTO* (Rigaku, 2006 ▸), *SIR92* (Altomare *et al.*, 1994 ▸), *SHELXS97* (Sheldrick, 2008 ▸), *SHELXL2014*/7 (Sheldrick, 2015 ▸), *ORTEP-3 for Windows* (Farrugia, 2012 ▸), *CrystalStructure* (Rigaku, 2010 ▸) and *PLATON* (Spek, 2009 ▸).

## supplementary crystallographic information

### (I) 4-Ethoxybenzoic acid–4,4'-bipyridyl (2/1) Crystal data

**Table:**

2C_9_H_10_O_3_·C_10_H_8_N_2_	*F*(000) = 1032.00
*M**_r_* = 488.52	*D*_x_ = 1.356 Mg m^−^^3^
Monoclinic, *P*2_1_/*n*	Mo *K*α radiation, λ = 0.71075 Å
*a* = 9.1090 (2) Å	Cell parameters from 23666 reflections
*b* = 20.9348 (5) Å	θ = 3.0–30.0°
*c* = 12.8738 (4) Å	µ = 0.10 mm^−^^1^
β = 102.9429 (10)°	*T* = 93 K
*V* = 2392.60 (11) Å^3^	Needle, colorless
*Z* = 4	0.28 × 0.25 × 0.10 mm

### (I) 4-Ethoxybenzoic acid–4,4'-bipyridyl (2/1) Data collection

**Table:**

Rigaku R-AXIS RAPIDII diffractometer	*R*_int_ = 0.035
Detector resolution: 10.000 pixels mm^-1^	θ_max_ = 30.0°
ω scans	*h* = −12→12
28629 measured reflections	*k* = −28→29
6941 independent reflections	*l* = −18→18
6004 reflections with *I* > 2σ(*I*)	

### (I) 4-Ethoxybenzoic acid–4,4'-bipyridyl (2/1) Refinement

**Table:**

Refinement on *F*^2^	Primary atom site location: structure-invariant direct methods
Least-squares matrix: full	Secondary atom site location: difference Fourier map
*R*[*F*^2^ > 2σ(*F*^2^)] = 0.045	Hydrogen site location: mixed
*wR*(*F*^2^) = 0.127	H atoms treated by a mixture of independent and constrained refinement
*S* = 1.04	*w* = 1/[σ^2^(*F*_o_^2^) + (0.0817*P*)^2^ + 0.3767*P*] where *P* = (*F*_o_^2^ + 2*F*_c_^2^)/3
6941 reflections	(Δ/σ)_max_ = 0.001
335 parameters	Δρ_max_ = 0.39 e Å^−^^3^
0 restraints	Δρ_min_ = −0.37 e Å^−^^3^

### (I) 4-Ethoxybenzoic acid–4,4'-bipyridyl (2/1) Special details

**Table:**

Geometry. All esds (except the esd in the dihedral angle between two l.s. planes) are estimated using the full covariance matrix. The cell esds are taken into account individually in the estimation of esds in distances, angles and torsion angles; correlations between esds in cell parameters are only used when they are defined by crystal symmetry. An approximate (isotropic) treatment of cell esds is used for estimating esds involving l.s. planes.
Refinement. Reflections were merged by SHELXL according to the crystal class for the calculation of statistics and refinement.

### (I) 4-Ethoxybenzoic acid–4,4'-bipyridyl (2/1) Fractional atomic coordinates and isotropic or equivalent isotropic displacement parameters (Å^2^)

**Table:**

	*x*	*y*	*z*	*U*_iso_*/*U*_eq_	
O1	−0.33398 (8)	0.30866 (3)	0.77777 (6)	0.02333 (16)	
O2	−0.37344 (8)	0.40959 (3)	0.72097 (6)	0.02177 (15)	
O3	−0.92113 (7)	0.36336 (3)	0.94659 (6)	0.01856 (14)	
O4	0.74748 (8)	0.35124 (3)	0.34692 (6)	0.02249 (16)	
O5	0.77838 (8)	0.45585 (3)	0.38227 (6)	0.02363 (16)	
O6	1.33565 (7)	0.38813 (3)	0.17479 (6)	0.01967 (15)	
N1	−0.10882 (9)	0.31350 (4)	0.67751 (7)	0.02016 (17)	
N2	0.51947 (9)	0.35033 (4)	0.44136 (7)	0.02191 (17)	
C1	−0.54350 (10)	0.36269 (4)	0.81521 (7)	0.01557 (17)	
C2	−0.57223 (10)	0.31282 (4)	0.88043 (7)	0.01700 (17)	
H2	−0.5048	0.2777	0.8950	0.020*	
C3	−0.69860 (10)	0.31438 (4)	0.92393 (8)	0.01764 (17)	
H3	−0.7169	0.2806	0.9686	0.021*	
C4	−0.79903 (10)	0.36588 (4)	0.90180 (7)	0.01553 (17)	
C5	−0.77115 (10)	0.41585 (4)	0.83675 (7)	0.01658 (17)	
H5	−0.8388	0.4509	0.8216	0.020*	
C6	−0.64371 (10)	0.41374 (4)	0.79445 (7)	0.01672 (17)	
H6	−0.6246	0.4478	0.7505	0.020*	
C7	−0.40997 (10)	0.36318 (4)	0.76677 (7)	0.01667 (17)	
C8	−1.02432 (10)	0.41622 (4)	0.92520 (8)	0.01880 (18)	
H8A	−1.0723	0.4179	0.8482	0.023*	
H8B	−0.9696	0.4568	0.9453	0.023*	
C9	−1.14267 (11)	0.40745 (5)	0.98921 (8)	0.02217 (19)	
H9A	−1.0945	0.4070	1.0653	0.033*	
H9B	−1.1954	0.3669	0.9695	0.033*	
H9C	−1.2150	0.4427	0.9744	0.033*	
C10	0.95579 (10)	0.40171 (4)	0.30216 (7)	0.01621 (17)	
C11	1.04428 (10)	0.45571 (4)	0.30003 (8)	0.01774 (17)	
H11	1.0160	0.4949	0.3273	0.021*	
C12	1.17334 (10)	0.45373 (4)	0.25886 (8)	0.01782 (18)	
H12	1.2328	0.4910	0.2585	0.021*	
C13	1.21379 (10)	0.39607 (4)	0.21808 (7)	0.01639 (17)	
C14	1.12647 (10)	0.34139 (4)	0.22033 (8)	0.01837 (18)	
H14	1.1544	0.3022	0.1928	0.022*	
C15	0.99956 (10)	0.34406 (4)	0.26245 (8)	0.01753 (17)	
H15	0.9416	0.3065	0.2645	0.021*	
C16	0.81942 (10)	0.40630 (5)	0.34748 (7)	0.01747 (17)	
C17	1.43238 (10)	0.44212 (5)	0.17318 (8)	0.02026 (19)	
H17A	1.4830	0.4543	0.2468	0.024*	
H17B	1.3729	0.4791	0.1388	0.024*	
C18	1.54764 (11)	0.42313 (5)	0.11099 (8)	0.0229 (2)	
H18A	1.5998	0.3843	0.1421	0.034*	
H18B	1.6209	0.4578	0.1139	0.034*	
H18C	1.4970	0.4150	0.0366	0.034*	
C19	−0.09136 (10)	0.36667 (5)	0.62319 (8)	0.01935 (18)	
H19	−0.1662	0.3990	0.6159	0.023*	
C20	0.03056 (10)	0.37669 (5)	0.57728 (7)	0.01765 (17)	
H20	0.0388	0.4153	0.5402	0.021*	
C21	0.14130 (10)	0.32950 (4)	0.58603 (7)	0.01586 (17)	
C22	0.12277 (10)	0.27418 (5)	0.64248 (8)	0.01962 (18)	
H22	0.1954	0.2409	0.6508	0.024*	
C23	−0.00263 (11)	0.26821 (5)	0.68641 (8)	0.02095 (19)	
H23	−0.0137	0.2303	0.7245	0.025*	
C24	0.46423 (11)	0.29264 (5)	0.45714 (9)	0.0242 (2)	
H24	0.5105	0.2559	0.4352	0.029*	
C25	0.34301 (11)	0.28424 (5)	0.50393 (9)	0.0228 (2)	
H25	0.3080	0.2424	0.5140	0.027*	
C26	0.27224 (10)	0.33746 (4)	0.53636 (7)	0.01618 (17)	
C27	0.32923 (10)	0.39752 (4)	0.51958 (8)	0.01849 (18)	
H27	0.2847	0.4352	0.5400	0.022*	
C28	0.45223 (11)	0.40137 (5)	0.47250 (8)	0.02086 (19)	
H28	0.4906	0.4425	0.4619	0.025*	
H1	−0.254 (2)	0.3118 (9)	0.7426 (17)	0.059 (5)*	
H4	0.663 (2)	0.3549 (10)	0.3782 (17)	0.066 (6)*	

### (I) 4-Ethoxybenzoic acid–4,4'-bipyridyl (2/1) Atomic displacement parameters (Å^2^)

**Table:**

	*U*^11^	*U*^22^	*U*^33^	*U*^12^	*U*^13^	*U*^23^
O1	0.0216 (3)	0.0188 (3)	0.0345 (4)	0.0023 (3)	0.0167 (3)	0.0000 (3)
O2	0.0200 (3)	0.0207 (3)	0.0274 (4)	−0.0013 (3)	0.0114 (3)	0.0013 (3)
O3	0.0148 (3)	0.0197 (3)	0.0238 (3)	0.0017 (2)	0.0099 (2)	0.0022 (2)
O4	0.0194 (3)	0.0226 (3)	0.0293 (4)	−0.0033 (3)	0.0136 (3)	−0.0007 (3)
O5	0.0217 (3)	0.0227 (3)	0.0293 (4)	0.0038 (3)	0.0117 (3)	−0.0003 (3)
O6	0.0165 (3)	0.0182 (3)	0.0276 (4)	−0.0019 (2)	0.0120 (3)	−0.0021 (3)
N1	0.0170 (3)	0.0235 (4)	0.0220 (4)	−0.0013 (3)	0.0086 (3)	−0.0038 (3)
N2	0.0168 (3)	0.0281 (4)	0.0228 (4)	0.0005 (3)	0.0088 (3)	0.0003 (3)
C1	0.0141 (4)	0.0167 (4)	0.0167 (4)	−0.0013 (3)	0.0053 (3)	−0.0030 (3)
C2	0.0152 (4)	0.0166 (4)	0.0198 (4)	0.0006 (3)	0.0052 (3)	−0.0012 (3)
C3	0.0163 (4)	0.0169 (4)	0.0207 (4)	−0.0003 (3)	0.0062 (3)	0.0019 (3)
C4	0.0134 (4)	0.0176 (4)	0.0165 (4)	−0.0009 (3)	0.0054 (3)	−0.0019 (3)
C5	0.0161 (4)	0.0157 (4)	0.0189 (4)	0.0009 (3)	0.0059 (3)	−0.0003 (3)
C6	0.0173 (4)	0.0165 (4)	0.0175 (4)	−0.0010 (3)	0.0061 (3)	−0.0007 (3)
C7	0.0150 (4)	0.0179 (4)	0.0181 (4)	−0.0015 (3)	0.0058 (3)	−0.0043 (3)
C8	0.0169 (4)	0.0185 (4)	0.0228 (4)	0.0022 (3)	0.0082 (3)	−0.0003 (3)
C9	0.0175 (4)	0.0280 (5)	0.0230 (5)	0.0027 (4)	0.0087 (3)	0.0021 (4)
C10	0.0148 (4)	0.0179 (4)	0.0168 (4)	0.0010 (3)	0.0051 (3)	0.0023 (3)
C11	0.0177 (4)	0.0159 (4)	0.0209 (4)	0.0019 (3)	0.0070 (3)	0.0005 (3)
C12	0.0164 (4)	0.0156 (4)	0.0227 (4)	−0.0010 (3)	0.0070 (3)	0.0012 (3)
C13	0.0142 (4)	0.0176 (4)	0.0183 (4)	0.0008 (3)	0.0056 (3)	0.0018 (3)
C14	0.0179 (4)	0.0150 (4)	0.0240 (4)	0.0009 (3)	0.0084 (3)	−0.0001 (3)
C15	0.0171 (4)	0.0159 (4)	0.0209 (4)	−0.0009 (3)	0.0069 (3)	0.0016 (3)
C16	0.0153 (4)	0.0207 (4)	0.0172 (4)	0.0015 (3)	0.0053 (3)	0.0026 (3)
C17	0.0181 (4)	0.0192 (4)	0.0260 (5)	−0.0034 (3)	0.0104 (4)	−0.0015 (3)
C18	0.0187 (4)	0.0272 (5)	0.0255 (5)	−0.0047 (4)	0.0108 (4)	−0.0055 (4)
C19	0.0159 (4)	0.0230 (4)	0.0201 (4)	0.0026 (3)	0.0061 (3)	−0.0022 (3)
C20	0.0159 (4)	0.0205 (4)	0.0174 (4)	0.0009 (3)	0.0057 (3)	−0.0007 (3)
C21	0.0134 (4)	0.0196 (4)	0.0153 (4)	−0.0008 (3)	0.0047 (3)	−0.0034 (3)
C22	0.0173 (4)	0.0200 (4)	0.0233 (4)	0.0022 (3)	0.0084 (3)	0.0000 (3)
C23	0.0204 (4)	0.0212 (4)	0.0237 (4)	−0.0006 (3)	0.0102 (4)	0.0007 (3)
C24	0.0212 (4)	0.0241 (4)	0.0313 (5)	0.0035 (4)	0.0141 (4)	−0.0014 (4)
C25	0.0218 (4)	0.0194 (4)	0.0313 (5)	0.0000 (4)	0.0147 (4)	−0.0019 (4)
C26	0.0139 (4)	0.0197 (4)	0.0160 (4)	0.0009 (3)	0.0055 (3)	−0.0010 (3)
C27	0.0186 (4)	0.0194 (4)	0.0191 (4)	0.0006 (3)	0.0077 (3)	−0.0007 (3)
C28	0.0201 (4)	0.0227 (4)	0.0218 (4)	−0.0028 (3)	0.0090 (3)	0.0000 (3)

### (I) 4-Ethoxybenzoic acid–4,4'-bipyridyl (2/1) Geometric parameters (Å, º)

**Table:**

O1—C7	1.3259 (11)	C10—C16	1.4891 (12)
O1—H1	0.942 (19)	C11—C12	1.3941 (12)
O2—C7	1.2208 (12)	C11—H11	0.9500
O3—C4	1.3637 (10)	C12—C13	1.3984 (13)
O3—C8	1.4380 (11)	C12—H12	0.9500
O4—C16	1.3252 (12)	C13—C14	1.3979 (12)
O4—H4	0.948 (19)	C14—C15	1.3836 (12)
O5—C16	1.2213 (12)	C14—H14	0.9500
O6—C13	1.3592 (10)	C15—H15	0.9500
O6—C17	1.4361 (11)	C17—C18	1.5092 (13)
N1—C23	1.3409 (13)	C17—H17A	0.9900
N1—C19	1.3429 (13)	C17—H17B	0.9900
N2—C28	1.3370 (13)	C18—H18A	0.9800
N2—C24	1.3412 (14)	C18—H18B	0.9800
C1—C6	1.3920 (12)	C18—H18C	0.9800
C1—C2	1.4006 (13)	C19—C20	1.3859 (12)
C1—C7	1.4860 (12)	C19—H19	0.9500
C2—C3	1.3887 (12)	C20—C21	1.3983 (12)
C2—H2	0.9500	C20—H20	0.9500
C3—C4	1.4012 (12)	C21—C22	1.3974 (13)
C3—H3	0.9500	C21—C26	1.4835 (12)
C4—C5	1.3984 (12)	C22—C23	1.3892 (12)
C5—C6	1.3890 (12)	C22—H22	0.9500
C5—H5	0.9500	C23—H23	0.9500
C6—H6	0.9500	C24—C25	1.3825 (13)
C8—C9	1.5077 (13)	C24—H24	0.9500
C8—H8A	0.9900	C25—C26	1.3971 (13)
C8—H8B	0.9900	C25—H25	0.9500
C9—H9A	0.9800	C26—C27	1.3956 (13)
C9—H9B	0.9800	C27—C28	1.3905 (12)
C9—H9C	0.9800	C27—H27	0.9500
C10—C11	1.3923 (12)	C28—H28	0.9500
C10—C15	1.4030 (12)		
			
C7—O1—H1	109.2 (12)	C15—C14—C13	120.28 (8)
C4—O3—C8	116.70 (7)	C15—C14—H14	119.9
C16—O4—H4	112.0 (13)	C13—C14—H14	119.9
C13—O6—C17	118.02 (7)	C14—C15—C10	120.54 (8)
C23—N1—C19	117.60 (8)	C14—C15—H15	119.7
C28—N2—C24	117.50 (8)	C10—C15—H15	119.7
C6—C1—C2	119.00 (8)	O5—C16—O4	123.31 (8)
C6—C1—C7	118.51 (8)	O5—C16—C10	123.32 (8)
C2—C1—C7	122.49 (8)	O4—C16—C10	113.37 (8)
C3—C2—C1	120.48 (8)	O6—C17—C18	107.60 (8)
C3—C2—H2	119.8	O6—C17—H17A	110.2
C1—C2—H2	119.8	C18—C17—H17A	110.2
C2—C3—C4	119.90 (8)	O6—C17—H17B	110.2
C2—C3—H3	120.0	C18—C17—H17B	110.2
C4—C3—H3	120.0	H17A—C17—H17B	108.5
O3—C4—C5	123.84 (8)	C17—C18—H18A	109.5
O3—C4—C3	116.20 (8)	C17—C18—H18B	109.5
C5—C4—C3	119.96 (8)	H18A—C18—H18B	109.5
C6—C5—C4	119.40 (8)	C17—C18—H18C	109.5
C6—C5—H5	120.3	H18A—C18—H18C	109.5
C4—C5—H5	120.3	H18B—C18—H18C	109.5
C5—C6—C1	121.25 (8)	N1—C19—C20	123.18 (8)
C5—C6—H6	119.4	N1—C19—H19	118.4
C1—C6—H6	119.4	C20—C19—H19	118.4
O2—C7—O1	123.00 (8)	C19—C20—C21	119.42 (9)
O2—C7—C1	123.05 (8)	C19—C20—H20	120.3
O1—C7—C1	113.95 (8)	C21—C20—H20	120.3
O3—C8—C9	108.56 (8)	C22—C21—C20	117.29 (8)
O3—C8—H8A	110.0	C22—C21—C26	121.30 (8)
C9—C8—H8A	110.0	C20—C21—C26	121.41 (8)
O3—C8—H8B	110.0	C23—C22—C21	119.50 (8)
C9—C8—H8B	110.0	C23—C22—H22	120.2
H8A—C8—H8B	108.4	C21—C22—H22	120.2
C8—C9—H9A	109.5	N1—C23—C22	123.01 (9)
C8—C9—H9B	109.5	N1—C23—H23	118.5
H9A—C9—H9B	109.5	C22—C23—H23	118.5
C8—C9—H9C	109.5	N2—C24—C25	122.95 (9)
H9A—C9—H9C	109.5	N2—C24—H24	118.5
H9B—C9—H9C	109.5	C25—C24—H24	118.5
C11—C10—C15	118.58 (8)	C24—C25—C26	119.73 (9)
C11—C10—C16	119.52 (8)	C24—C25—H25	120.1
C15—C10—C16	121.89 (8)	C26—C25—H25	120.1
C10—C11—C12	121.64 (8)	C27—C26—C25	117.37 (8)
C10—C11—H11	119.2	C27—C26—C21	122.05 (8)
C12—C11—H11	119.2	C25—C26—C21	120.57 (8)
C11—C12—C13	118.94 (8)	C28—C27—C26	118.92 (8)
C11—C12—H12	120.5	C28—C27—H27	120.5
C13—C12—H12	120.5	C26—C27—H27	120.5
O6—C13—C14	115.41 (8)	N2—C28—C27	123.53 (9)
O6—C13—C12	124.58 (8)	N2—C28—H28	118.2
C14—C13—C12	120.01 (8)	C27—C28—H28	118.2
			
C6—C1—C2—C3	−0.16 (13)	C11—C10—C15—C14	−1.18 (14)
C7—C1—C2—C3	−179.95 (8)	C16—C10—C15—C14	179.68 (8)
C1—C2—C3—C4	0.57 (14)	C11—C10—C16—O5	1.11 (14)
C8—O3—C4—C5	−0.72 (13)	C15—C10—C16—O5	−179.75 (9)
C8—O3—C4—C3	179.48 (8)	C11—C10—C16—O4	−178.52 (8)
C2—C3—C4—O3	179.25 (8)	C15—C10—C16—O4	0.62 (13)
C2—C3—C4—C5	−0.56 (14)	C13—O6—C17—C18	−173.89 (8)
O3—C4—C5—C6	−179.65 (8)	C23—N1—C19—C20	−0.45 (14)
C3—C4—C5—C6	0.14 (14)	N1—C19—C20—C21	0.69 (14)
C4—C5—C6—C1	0.28 (14)	C19—C20—C21—C22	−0.57 (13)
C2—C1—C6—C5	−0.27 (14)	C19—C20—C21—C26	178.73 (8)
C7—C1—C6—C5	179.53 (8)	C20—C21—C22—C23	0.28 (14)
C6—C1—C7—O2	9.58 (13)	C26—C21—C22—C23	−179.03 (9)
C2—C1—C7—O2	−170.62 (9)	C19—N1—C23—C22	0.13 (15)
C6—C1—C7—O1	−170.43 (8)	C21—C22—C23—N1	−0.05 (15)
C2—C1—C7—O1	9.37 (13)	C28—N2—C24—C25	−0.27 (16)
C4—O3—C8—C9	−174.93 (8)	N2—C24—C25—C26	0.41 (17)
C15—C10—C11—C12	0.53 (14)	C24—C25—C26—C27	−0.13 (15)
C16—C10—C11—C12	179.70 (8)	C24—C25—C26—C21	179.56 (9)
C10—C11—C12—C13	0.48 (14)	C22—C21—C26—C27	−152.77 (9)
C17—O6—C13—C14	−178.14 (8)	C20—C21—C26—C27	27.95 (13)
C17—O6—C13—C12	1.77 (13)	C22—C21—C26—C25	27.55 (13)
C11—C12—C13—O6	179.23 (9)	C20—C21—C26—C25	−151.72 (10)
C11—C12—C13—C14	−0.87 (14)	C25—C26—C27—C28	−0.26 (14)
O6—C13—C14—C15	−179.85 (8)	C21—C26—C27—C28	−179.94 (9)
C12—C13—C14—C15	0.24 (14)	C24—N2—C28—C27	−0.16 (15)
C13—C14—C15—C10	0.80 (14)	C26—C27—C28—N2	0.43 (15)

### (I) 4-Ethoxybenzoic acid–4,4'-bipyridyl (2/1) Hydrogen-bond geometry (Å, º)

*Cg*1 and *Cg*2 are the centroids of the C1–C6 and C10–C15 rings, respectively.

**Table:**

*D*—H···*A*	*D*—H	H···*A*	*D*···*A*	*D*—H···*A*
O1—H1···N1	0.942 (19)	1.72 (2)	2.6587 (11)	177.2 (19)
O4—H4···N2	0.948 (19)	1.690 (19)	2.6312 (11)	171.4 (19)
C12—H12···O2^i^	0.95	2.43	3.3712 (11)	172
C14—H14···O1^ii^	0.95	2.56	3.2288 (11)	128
C24—H24···O3^ii^	0.95	2.57	3.4407 (12)	153
C9—H9*A*···*Cg*2^iii^	0.98	2.68	3.6450 (11)	169
C18—H18*C*···*Cg*1^iv^	0.98	2.67	3.6253 (11)	164

Symmetry codes: (i) −*x*+1, −*y*+1, −*z*+1; (ii) *x*+3/2, −*y*+1/2, *z*−1/2; (iii) *x*−2, *y*, *z*+1; (iv) *x*+2, *y*, *z*−1.

### (II) 4-n-Propoxybenzoic acid–4,4'-bipyridyl (2/1) Crystal data

**Table:**

2C_10_H_12_O_3_·C_10_H_8_N_2_	*Z* = 2
*M**_r_* = 516.57	*F*(000) = 548.00
Triclinic, *P*1	*D*_x_ = 1.312 Mg m^−^^3^
*a* = 10.7592 (4) Å	Mo *K*α radiation, λ = 0.71075 Å
*b* = 10.8838 (3) Å	Cell parameters from 13551 reflections
*c* = 11.6462 (4) Å	θ = 3.0–30.0°
α = 86.6411 (11)°	µ = 0.09 mm^−^^1^
β = 89.2313 (13)°	*T* = 93 K
γ = 73.8867 (12)°	Block, colorless
*V* = 1307.95 (8) Å^3^	0.50 × 0.40 × 0.10 mm

### (II) 4-n-Propoxybenzoic acid–4,4'-bipyridyl (2/1) Data collection

**Table:**

Rigaku R-AXIS RAPIDII diffractometer	*R*_int_ = 0.069
Detector resolution: 10.000 pixels mm^-1^	θ_max_ = 30.0°, θ_min_ = 3.0°
ω scans	*h* = −15→15
15909 measured reflections	*k* = −13→15
7507 independent reflections	*l* = −16→16
5980 reflections with *I* > 2σ(*I*)	

### (II) 4-n-Propoxybenzoic acid–4,4'-bipyridyl (2/1) Refinement

**Table:**

Refinement on *F*^2^	Secondary atom site location: difference Fourier map
Least-squares matrix: full	Hydrogen site location: mixed
*R*[*F*^2^ > 2σ(*F*^2^)] = 0.047	H atoms treated by a mixture of independent and constrained refinement
*wR*(*F*^2^) = 0.135	*w* = 1/[σ^2^(*F*_o_^2^) + (0.090*P*)^2^] where *P* = (*F*_o_^2^ + 2*F*_c_^2^)/3
*S* = 1.04	(Δ/σ)_max_ = 0.001
7507 reflections	Δρ_max_ = 0.35 e Å^−^^3^
354 parameters	Δρ_min_ = −0.32 e Å^−^^3^
0 restraints	Extinction correction: SHELXL-2014/7, Fc^*^=kFc[1+0.001xFc^2^λ^3^/sin(2θ)]^-1/4^
Primary atom site location: structure-invariant direct methods	Extinction coefficient: 0.019 (4)

### (II) 4-n-Propoxybenzoic acid–4,4'-bipyridyl (2/1) Special details

**Table:**

Geometry. All esds (except the esd in the dihedral angle between two l.s. planes) are estimated using the full covariance matrix. The cell esds are taken into account individually in the estimation of esds in distances, angles and torsion angles; correlations between esds in cell parameters are only used when they are defined by crystal symmetry. An approximate (isotropic) treatment of cell esds is used for estimating esds involving l.s. planes.
Refinement. Reflections were merged by SHELXL according to the crystal class for the calculation of statistics and refinement.

### (II) 4-n-Propoxybenzoic acid–4,4'-bipyridyl (2/1) Fractional atomic coordinates and isotropic or equivalent isotropic displacement parameters (Å^2^)

**Table:**

	*x*	*y*	*z*	*U*_iso_*/*U*_eq_	
O1	0.21741 (6)	0.84171 (6)	−0.00993 (6)	0.02370 (16)	
O2	0.26015 (7)	0.62711 (6)	0.01082 (6)	0.02497 (16)	
O3	−0.31475 (6)	0.79522 (6)	−0.19102 (6)	0.02084 (15)	
O4	1.32849 (7)	0.66282 (6)	0.36794 (6)	0.02574 (16)	
O5	1.28863 (7)	0.87599 (6)	0.33722 (6)	0.02456 (15)	
O6	1.86048 (6)	0.70388 (6)	0.55057 (5)	0.01982 (14)	
N1	0.44816 (7)	0.80971 (7)	0.08178 (6)	0.01957 (16)	
N2	1.08835 (7)	0.69161 (7)	0.29159 (6)	0.01975 (16)	
C1	0.05619 (8)	0.74854 (8)	−0.06643 (7)	0.01587 (16)	
C2	−0.02538 (8)	0.86942 (8)	−0.09987 (7)	0.01713 (16)	
H2	0.0038	0.9435	−0.0934	0.021*	
C3	−0.14819 (8)	0.88217 (8)	−0.14234 (7)	0.01796 (17)	
H3	−0.2024	0.9644	−0.1660	0.022*	
C4	−0.19207 (8)	0.77306 (8)	−0.15022 (7)	0.01676 (17)	
C5	−0.11202 (8)	0.65191 (8)	−0.11692 (7)	0.01732 (16)	
H5	−0.1416	0.5779	−0.1222	0.021*	
C6	0.01143 (8)	0.64099 (8)	−0.07598 (7)	0.01671 (16)	
H6	0.0665	0.5586	−0.0541	0.020*	
C7	0.18746 (8)	0.73163 (8)	−0.01851 (7)	0.01780 (17)	
C8	−0.36390 (8)	0.68649 (8)	−0.20224 (7)	0.01802 (17)	
H8A	−0.3609	0.6385	−0.1270	0.022*	
H8B	−0.3108	0.6283	−0.2576	0.022*	
C9	−0.50170 (9)	0.73524 (9)	−0.24460 (8)	0.02173 (18)	
H9B	−0.5034	0.7767	−0.3228	0.026*	
H9A	−0.5522	0.7999	−0.1931	0.026*	
C10	−0.56236 (9)	0.62384 (9)	−0.24724 (10)	0.0281 (2)	
H10A	−0.6465	0.6532	−0.2862	0.042*	
H10B	−0.5742	0.5925	−0.1684	0.042*	
H10C	−0.5053	0.5544	−0.2889	0.042*	
C11	1.49360 (8)	0.75522 (8)	0.41394 (7)	0.01674 (16)	
C12	1.57341 (9)	0.63400 (8)	0.44879 (7)	0.01858 (17)	
H12	1.5434	0.5605	0.4418	0.022*	
C13	1.69517 (9)	0.62010 (8)	0.49316 (7)	0.01892 (17)	
H13	1.7485	0.5373	0.5164	0.023*	
C14	1.74023 (8)	0.72791 (8)	0.50403 (7)	0.01712 (17)	
C15	1.66235 (9)	0.84947 (8)	0.46796 (7)	0.01804 (17)	
H15	1.6927	0.9229	0.4740	0.022*	
C16	1.54008 (8)	0.86179 (8)	0.42315 (7)	0.01771 (17)	
H16	1.4873	0.9443	0.3984	0.021*	
C17	1.36100 (9)	0.77237 (8)	0.36890 (7)	0.01841 (17)	
C18	1.91667 (8)	0.80929 (8)	0.55338 (7)	0.01815 (17)	
H18A	1.8635	0.8761	0.6016	0.022*	
H18B	1.9206	0.8477	0.4747	0.022*	
C19	2.05137 (9)	0.75818 (9)	0.60313 (8)	0.02037 (18)	
H19B	2.1028	0.6888	0.5564	0.024*	
H19A	2.0465	0.7220	0.6825	0.024*	
C20	2.11746 (9)	0.86578 (9)	0.60422 (9)	0.0277 (2)	
H20A	2.0643	0.9360	0.6475	0.042*	
H20B	2.1277	0.8972	0.5251	0.042*	
H20C	2.2027	0.8330	0.6408	0.042*	
C21	0.52853 (9)	0.69053 (9)	0.08870 (8)	0.02069 (18)	
H21	0.4970	0.6220	0.0666	0.025*	
C22	0.65531 (8)	0.66285 (8)	0.12658 (8)	0.01907 (17)	
H22	0.7088	0.5771	0.1302	0.023*	
C23	0.70378 (8)	0.76194 (8)	0.15928 (7)	0.01608 (16)	
C24	0.61977 (8)	0.88583 (8)	0.15197 (7)	0.01863 (17)	
H24	0.6488	0.9563	0.1730	0.022*	
C25	0.49411 (8)	0.90534 (8)	0.11387 (7)	0.01989 (17)	
H25	0.4380	0.9900	0.1103	0.024*	
C26	1.02841 (8)	0.80376 (8)	0.23616 (7)	0.02020 (18)	
H26	1.0730	0.8678	0.2273	0.024*	
C27	0.90438 (8)	0.83033 (8)	0.19124 (7)	0.01839 (17)	
H27	0.8655	0.9110	0.1527	0.022*	
C28	0.83731 (8)	0.73745 (8)	0.20317 (7)	0.01565 (16)	
C29	0.90043 (8)	0.62059 (8)	0.26043 (7)	0.01836 (17)	
H29	0.8586	0.5544	0.2701	0.022*	
C30	1.02416 (9)	0.60203 (8)	0.30285 (7)	0.02023 (18)	
H30	1.0656	0.5222	0.3417	0.024*	
H1	0.305 (2)	0.8292 (19)	0.0308 (18)	0.088 (6)*	
H4	1.240 (2)	0.6740 (19)	0.3337 (16)	0.082 (6)*	

### (II) 4-n-Propoxybenzoic acid–4,4'-bipyridyl (2/1) Atomic displacement parameters (Å^2^)

**Table:**

	*U*^11^	*U*^22^	*U*^33^	*U*^12^	*U*^13^	*U*^23^
O1	0.0188 (3)	0.0171 (3)	0.0356 (4)	−0.0057 (3)	−0.0076 (3)	0.0000 (3)
O2	0.0195 (3)	0.0176 (3)	0.0355 (4)	−0.0016 (2)	−0.0077 (3)	0.0014 (3)
O3	0.0148 (3)	0.0155 (3)	0.0318 (3)	−0.0040 (2)	−0.0071 (2)	0.0017 (2)
O4	0.0198 (3)	0.0180 (3)	0.0396 (4)	−0.0060 (3)	−0.0084 (3)	0.0027 (3)
O5	0.0202 (3)	0.0193 (3)	0.0314 (3)	−0.0018 (3)	−0.0051 (3)	0.0043 (3)
O6	0.0175 (3)	0.0163 (3)	0.0255 (3)	−0.0048 (2)	−0.0064 (2)	0.0022 (2)
N1	0.0156 (3)	0.0211 (4)	0.0216 (3)	−0.0048 (3)	−0.0021 (3)	0.0009 (3)
N2	0.0166 (3)	0.0199 (3)	0.0219 (3)	−0.0035 (3)	−0.0022 (3)	−0.0019 (3)
C1	0.0154 (4)	0.0154 (4)	0.0163 (3)	−0.0036 (3)	−0.0007 (3)	0.0006 (3)
C2	0.0171 (4)	0.0139 (3)	0.0207 (4)	−0.0049 (3)	−0.0016 (3)	0.0000 (3)
C3	0.0165 (4)	0.0132 (3)	0.0229 (4)	−0.0025 (3)	−0.0028 (3)	0.0018 (3)
C4	0.0143 (4)	0.0161 (4)	0.0188 (4)	−0.0028 (3)	−0.0019 (3)	0.0007 (3)
C5	0.0178 (4)	0.0139 (4)	0.0205 (4)	−0.0049 (3)	−0.0018 (3)	0.0003 (3)
C6	0.0168 (4)	0.0134 (3)	0.0186 (3)	−0.0024 (3)	−0.0017 (3)	0.0012 (3)
C7	0.0167 (4)	0.0173 (4)	0.0190 (4)	−0.0041 (3)	−0.0011 (3)	−0.0008 (3)
C8	0.0172 (4)	0.0164 (4)	0.0206 (4)	−0.0051 (3)	−0.0024 (3)	0.0003 (3)
C9	0.0169 (4)	0.0194 (4)	0.0287 (4)	−0.0052 (3)	−0.0052 (3)	0.0013 (3)
C10	0.0205 (4)	0.0241 (4)	0.0409 (5)	−0.0080 (4)	−0.0064 (4)	−0.0010 (4)
C11	0.0161 (4)	0.0166 (4)	0.0168 (3)	−0.0036 (3)	−0.0004 (3)	0.0003 (3)
C12	0.0196 (4)	0.0153 (4)	0.0209 (4)	−0.0052 (3)	−0.0017 (3)	0.0005 (3)
C13	0.0196 (4)	0.0136 (4)	0.0220 (4)	−0.0024 (3)	−0.0031 (3)	0.0017 (3)
C14	0.0163 (4)	0.0168 (4)	0.0170 (3)	−0.0027 (3)	−0.0011 (3)	0.0005 (3)
C15	0.0189 (4)	0.0146 (4)	0.0203 (4)	−0.0042 (3)	−0.0012 (3)	0.0000 (3)
C16	0.0177 (4)	0.0148 (4)	0.0187 (4)	−0.0016 (3)	−0.0006 (3)	0.0010 (3)
C17	0.0176 (4)	0.0185 (4)	0.0179 (4)	−0.0034 (3)	−0.0003 (3)	0.0006 (3)
C18	0.0178 (4)	0.0161 (4)	0.0206 (4)	−0.0051 (3)	−0.0021 (3)	0.0010 (3)
C19	0.0177 (4)	0.0193 (4)	0.0239 (4)	−0.0052 (3)	−0.0035 (3)	0.0017 (3)
C20	0.0213 (4)	0.0252 (4)	0.0376 (5)	−0.0091 (4)	−0.0056 (4)	0.0043 (4)
C21	0.0184 (4)	0.0204 (4)	0.0240 (4)	−0.0064 (3)	−0.0024 (3)	−0.0019 (3)
C22	0.0163 (4)	0.0164 (4)	0.0238 (4)	−0.0031 (3)	−0.0019 (3)	−0.0010 (3)
C23	0.0142 (4)	0.0172 (4)	0.0160 (3)	−0.0035 (3)	0.0002 (3)	0.0010 (3)
C24	0.0174 (4)	0.0158 (4)	0.0223 (4)	−0.0042 (3)	−0.0012 (3)	−0.0003 (3)
C25	0.0164 (4)	0.0180 (4)	0.0235 (4)	−0.0025 (3)	−0.0016 (3)	0.0025 (3)
C26	0.0171 (4)	0.0214 (4)	0.0227 (4)	−0.0066 (3)	−0.0014 (3)	0.0006 (3)
C27	0.0167 (4)	0.0167 (4)	0.0213 (4)	−0.0043 (3)	−0.0016 (3)	0.0018 (3)
C28	0.0138 (4)	0.0164 (4)	0.0161 (3)	−0.0030 (3)	0.0000 (3)	−0.0017 (3)
C29	0.0169 (4)	0.0157 (4)	0.0219 (4)	−0.0036 (3)	−0.0018 (3)	−0.0002 (3)
C30	0.0180 (4)	0.0175 (4)	0.0235 (4)	−0.0021 (3)	−0.0037 (3)	−0.0002 (3)

### (II) 4-n-Propoxybenzoic acid–4,4'-bipyridyl (2/1) Geometric parameters (Å, º)

**Table:**

O1—C7	1.3335 (10)	C11—C17	1.4853 (12)
O1—H1	1.03 (2)	C12—C13	1.3802 (12)
O2—C7	1.2208 (11)	C12—H12	0.9500
O3—C4	1.3612 (10)	C13—C14	1.4010 (11)
O3—C8	1.4368 (10)	C13—H13	0.9500
O4—C17	1.3331 (11)	C14—C15	1.3993 (12)
O4—H4	1.01 (2)	C15—C16	1.3904 (12)
O5—C17	1.2190 (11)	C15—H15	0.9500
O6—C14	1.3603 (10)	C16—H16	0.9500
O6—C18	1.4397 (10)	C18—C19	1.5109 (11)
N1—C25	1.3430 (11)	C18—H18A	0.9900
N1—C21	1.3430 (12)	C18—H18B	0.9900
N2—C30	1.3420 (11)	C19—C20	1.5301 (12)
N2—C26	1.3433 (12)	C19—H19B	0.9900
C1—C6	1.3944 (11)	C19—H19A	0.9900
C1—C2	1.4004 (12)	C20—H20A	0.9800
C1—C7	1.4848 (11)	C20—H20B	0.9800
C2—C3	1.3848 (11)	C20—H20C	0.9800
C2—H2	0.9500	C21—C22	1.3851 (12)
C3—C4	1.4023 (11)	C21—H21	0.9500
C3—H3	0.9500	C22—C23	1.3950 (11)
C4—C5	1.3963 (12)	C22—H22	0.9500
C5—C6	1.3886 (11)	C23—C24	1.3981 (11)
C5—H5	0.9500	C23—C28	1.4790 (11)
C6—H6	0.9500	C24—C25	1.3840 (12)
C8—C9	1.5084 (11)	C24—H24	0.9500
C8—H8A	0.9900	C25—H25	0.9500
C8—H8B	0.9900	C26—C27	1.3875 (11)
C9—C10	1.5301 (12)	C26—H26	0.9500
C9—H9B	0.9900	C27—C28	1.3955 (11)
C9—H9A	0.9900	C27—H27	0.9500
C10—H10A	0.9800	C28—C29	1.3990 (12)
C10—H10B	0.9800	C29—C30	1.3834 (11)
C10—H10C	0.9800	C29—H29	0.9500
C11—C16	1.3946 (11)	C30—H30	0.9500
C11—C12	1.4002 (12)		
			
C7—O1—H1	112.5 (11)	C16—C15—C14	119.37 (8)
C4—O3—C8	117.72 (6)	C16—C15—H15	120.3
C17—O4—H4	113.0 (11)	C14—C15—H15	120.3
C14—O6—C18	117.65 (6)	C15—C16—C11	121.16 (8)
C25—N1—C21	117.73 (7)	C15—C16—H16	119.4
C30—N2—C26	117.69 (7)	C11—C16—H16	119.4
C6—C1—C2	118.87 (7)	O5—C17—O4	123.34 (8)
C6—C1—C7	119.01 (7)	O5—C17—C11	123.60 (8)
C2—C1—C7	122.11 (7)	O4—C17—C11	113.06 (7)
C3—C2—C1	120.70 (7)	O6—C18—C19	107.89 (7)
C3—C2—H2	119.6	O6—C18—H18A	110.1
C1—C2—H2	119.6	C19—C18—H18A	110.1
C2—C3—C4	119.63 (7)	O6—C18—H18B	110.1
C2—C3—H3	120.2	C19—C18—H18B	110.1
C4—C3—H3	120.2	H18A—C18—H18B	108.4
O3—C4—C5	124.21 (8)	C18—C19—C20	110.05 (7)
O3—C4—C3	115.45 (7)	C18—C19—H19B	109.6
C5—C4—C3	120.34 (8)	C20—C19—H19B	109.6
C6—C5—C4	119.14 (8)	C18—C19—H19A	109.6
C6—C5—H5	120.4	C20—C19—H19A	109.6
C4—C5—H5	120.4	H19B—C19—H19A	108.2
C5—C6—C1	121.31 (8)	C19—C20—H20A	109.5
C5—C6—H6	119.3	C19—C20—H20B	109.5
C1—C6—H6	119.3	H20A—C20—H20B	109.5
O2—C7—O1	123.50 (8)	C19—C20—H20C	109.5
O2—C7—C1	123.11 (8)	H20A—C20—H20C	109.5
O1—C7—C1	113.39 (7)	H20B—C20—H20C	109.5
O3—C8—C9	107.90 (7)	N1—C21—C22	123.03 (8)
O3—C8—H8A	110.1	N1—C21—H21	118.5
C9—C8—H8A	110.1	C22—C21—H21	118.5
O3—C8—H8B	110.1	C21—C22—C23	119.47 (8)
C9—C8—H8B	110.1	C21—C22—H22	120.3
H8A—C8—H8B	108.4	C23—C22—H22	120.3
C8—C9—C10	109.79 (7)	C22—C23—C24	117.30 (8)
C8—C9—H9B	109.7	C22—C23—C28	121.74 (8)
C10—C9—H9B	109.7	C24—C23—C28	120.94 (8)
C8—C9—H9A	109.7	C25—C24—C23	119.67 (8)
C10—C9—H9A	109.7	C25—C24—H24	120.2
H9B—C9—H9A	108.2	C23—C24—H24	120.2
C9—C10—H10A	109.5	N1—C25—C24	122.80 (8)
C9—C10—H10B	109.5	N1—C25—H25	118.6
H10A—C10—H10B	109.5	C24—C25—H25	118.6
C9—C10—H10C	109.5	N2—C26—C27	123.00 (8)
H10A—C10—H10C	109.5	N2—C26—H26	118.5
H10B—C10—H10C	109.5	C27—C26—H26	118.5
C16—C11—C12	118.80 (8)	C26—C27—C28	119.40 (8)
C16—C11—C17	119.71 (7)	C26—C27—H27	120.3
C12—C11—C17	121.49 (8)	C28—C27—H27	120.3
C13—C12—C11	120.75 (8)	C27—C28—C29	117.37 (7)
C13—C12—H12	119.6	C27—C28—C23	121.57 (8)
C11—C12—H12	119.6	C29—C28—C23	121.05 (8)
C12—C13—C14	120.08 (8)	C30—C29—C28	119.54 (8)
C12—C13—H13	120.0	C30—C29—H29	120.2
C14—C13—H13	120.0	C28—C29—H29	120.2
O6—C14—C15	124.82 (7)	N2—C30—C29	123.00 (8)
O6—C14—C13	115.36 (7)	N2—C30—H30	118.5
C15—C14—C13	119.82 (8)	C29—C30—H30	118.5
			
C6—C1—C2—C3	−0.36 (13)	C12—C11—C16—C15	−1.18 (13)
C7—C1—C2—C3	−178.83 (7)	C17—C11—C16—C15	178.18 (7)
C1—C2—C3—C4	1.02 (13)	C16—C11—C17—O5	0.98 (13)
C8—O3—C4—C5	−1.09 (12)	C12—C11—C17—O5	−179.69 (8)
C8—O3—C4—C3	179.21 (7)	C16—C11—C17—O4	−178.26 (7)
C2—C3—C4—O3	178.88 (7)	C12—C11—C17—O4	1.08 (12)
C2—C3—C4—C5	−0.84 (13)	C14—O6—C18—C19	−177.34 (7)
O3—C4—C5—C6	−179.70 (7)	O6—C18—C19—C20	178.05 (7)
C3—C4—C5—C6	−0.01 (13)	C25—N1—C21—C22	−0.30 (13)
C4—C5—C6—C1	0.69 (13)	N1—C21—C22—C23	−0.11 (14)
C2—C1—C6—C5	−0.51 (13)	C21—C22—C23—C24	0.12 (12)
C7—C1—C6—C5	178.01 (7)	C21—C22—C23—C28	178.42 (8)
C6—C1—C7—O2	1.75 (13)	C22—C23—C24—C25	0.27 (12)
C2—C1—C7—O2	−179.78 (8)	C28—C23—C24—C25	−178.04 (8)
C6—C1—C7—O1	−177.75 (7)	C21—N1—C25—C24	0.72 (13)
C2—C1—C7—O1	0.72 (12)	C23—C24—C25—N1	−0.72 (13)
C4—O3—C8—C9	178.04 (7)	C30—N2—C26—C27	0.30 (13)
O3—C8—C9—C10	−174.77 (7)	N2—C26—C27—C28	−0.11 (13)
C16—C11—C12—C13	0.99 (13)	C26—C27—C28—C29	−0.23 (12)
C17—C11—C12—C13	−178.35 (8)	C26—C27—C28—C23	178.56 (8)
C11—C12—C13—C14	0.13 (13)	C22—C23—C28—C27	152.95 (9)
C18—O6—C14—C15	−5.96 (12)	C24—C23—C28—C27	−28.82 (12)
C18—O6—C14—C13	174.06 (7)	C22—C23—C28—C29	−28.31 (12)
C12—C13—C14—O6	178.89 (7)	C24—C23—C28—C29	149.93 (8)
C12—C13—C14—C15	−1.09 (13)	C27—C28—C29—C30	0.37 (12)
O6—C14—C15—C16	−179.08 (7)	C23—C28—C29—C30	−178.42 (8)
C13—C14—C15—C16	0.91 (13)	C26—N2—C30—C29	−0.15 (13)
C14—C15—C16—C11	0.23 (13)	C28—C29—C30—N2	−0.19 (13)

### (II) 4-n-Propoxybenzoic acid–4,4'-bipyridyl (2/1) Hydrogen-bond geometry (Å, º)

*Cg*1 and *Cg*2 are the centroids of the C1–C6 and C11–C16 rings, respectively.

**Table:**

*D*—H···*A*	*D*—H	H···*A*	*D*···*A*	*D*—H···*A*
O1—H1···N1	1.03 (2)	1.61 (2)	2.6407 (10)	174.3 (19)
O4—H4···N2	1.01 (2)	1.67 (2)	2.6728 (11)	173.9 (18)
C3—H3···O5^i^	0.95	2.57	3.3981 (11)	146
C25—H25···O3^ii^	0.95	2.57	3.4581 (11)	156
C9—H9*B*···*Cg*2^iii^	0.99	2.84	3.6750 (1)	142
C19—H19*A*···*Cg*1^iv^	0.99	2.72	3.5781 (1)	146

Symmetry codes: (i) −*x*+1, −*y*+2, −*z*; (ii) −*x*, −*y*+2, −*z*; (iii) *x*−2, *y*, *z*−1; (iv) *x*+2, *y*, *z*+1.

### (III) 4-n-Butoxybenzoic acid–4,4'-bipyridyl (2/1) Crystal data

**Table:**

2C_11_H_14_O_3_·C_10_H_8_N_2_	*Z* = 2
*M**_r_* = 544.63	*F*(000) = 580.00
Triclinic, *P*1	*D*_x_ = 1.264 Mg m^−^^3^
*a* = 7.6645 (10) Å	Mo *K*α radiation, λ = 0.71075 Å
*b* = 8.5087 (13) Å	Cell parameters from 9788 reflections
*c* = 22.606 (3) Å	θ = 3.2–30.1°
α = 80.498 (3)°	µ = 0.09 mm^−^^1^
β = 86.486 (3)°	*T* = 93 K
γ = 80.082 (3)°	Platelet, colorless
*V* = 1431.5 (4) Å^3^	0.53 × 0.41 × 0.11 mm

### (III) 4-n-Butoxybenzoic acid–4,4'-bipyridyl (2/1) Data collection

**Table:**

Rigaku R-AXIS RAPIDII diffractometer	*R*_int_ = 0.075
Detector resolution: 10.000 pixels mm^-1^	θ_max_ = 26.0°
ω scans	*h* = −8→9
12433 measured reflections	*k* = −10→10
5612 independent reflections	*l* = −27→27
3432 reflections with *I* > 2σ(*I*)	

### (III) 4-n-Butoxybenzoic acid–4,4'-bipyridyl (2/1) Refinement

**Table:**

Refinement on *F*^2^	Primary atom site location: structure-invariant direct methods
Least-squares matrix: full	Secondary atom site location: difference Fourier map
*R*[*F*^2^ > 2σ(*F*^2^)] = 0.069	Hydrogen site location: mixed
*wR*(*F*^2^) = 0.193	H atoms treated by a mixture of independent and constrained refinement
*S* = 1.01	*w* = 1/[σ^2^(*F*_o_^2^) + (0.0965*P*)^2^] where *P* = (*F*_o_^2^ + 2*F*_c_^2^)/3
5610 reflections	(Δ/σ)_max_ < 0.001
371 parameters	Δρ_max_ = 0.24 e Å^−^^3^
0 restraints	Δρ_min_ = −0.41 e Å^−^^3^

### (III) 4-n-Butoxybenzoic acid–4,4'-bipyridyl (2/1) Special details

**Table:**

Geometry. All esds (except the esd in the dihedral angle between two l.s. planes) are estimated using the full covariance matrix. The cell esds are taken into account individually in the estimation of esds in distances, angles and torsion angles; correlations between esds in cell parameters are only used when they are defined by crystal symmetry. An approximate (isotropic) treatment of cell esds is used for estimating esds involving l.s. planes.
Refinement. Reflections were merged by SHELXL according to the crystal class for the calculation of statistics and refinement.

### (III) 4-n-Butoxybenzoic acid–4,4'-bipyridyl (2/1) Fractional atomic coordinates and isotropic or equivalent isotropic displacement parameters (Å^2^)

**Table:**

	*x*	*y*	*z*	*U*_iso_*/*U*_eq_	
O1	0.1390 (2)	−0.1143 (2)	−0.18237 (8)	0.0358 (4)	
O2	0.3054 (2)	−0.3129 (2)	−0.12317 (8)	0.0399 (5)	
O3	0.1632 (2)	−0.6711 (2)	−0.33646 (8)	0.0347 (4)	
O4	0.2363 (2)	0.7005 (2)	0.21058 (8)	0.0380 (5)	
O5	0.3659 (3)	0.8609 (3)	0.13954 (8)	0.0433 (5)	
O6	0.3442 (2)	1.2321 (2)	0.36266 (7)	0.0336 (4)	
N1	0.1708 (3)	0.0497 (3)	−0.09736 (10)	0.0375 (5)	
N2	0.2511 (3)	0.5220 (3)	0.12499 (9)	0.0343 (5)	
C1	0.2120 (3)	−0.3677 (3)	−0.21459 (10)	0.0288 (5)	
C2	0.1061 (3)	−0.3154 (3)	−0.26457 (11)	0.0318 (6)	
H2	0.0429	−0.2080	−0.2710	0.038*	
C3	0.0926 (3)	−0.4186 (3)	−0.30463 (11)	0.0325 (6)	
H3	0.0206	−0.3821	−0.3385	0.039*	
C4	0.1843 (3)	−0.5760 (3)	−0.29530 (11)	0.0306 (5)	
C5	0.2917 (3)	−0.6282 (3)	−0.24626 (11)	0.0329 (6)	
H5	0.3555	−0.7354	−0.2399	0.040*	
C6	0.3055 (3)	−0.5231 (3)	−0.20661 (11)	0.0323 (6)	
H6	0.3804	−0.5586	−0.1734	0.039*	
C7	0.2243 (3)	−0.2636 (3)	−0.16939 (11)	0.0323 (6)	
C8	0.2479 (3)	−0.8367 (3)	−0.32529 (12)	0.0338 (6)	
H8A	0.2005	−0.8918	−0.2872	0.041*	
H8B	0.3771	−0.8427	−0.3219	0.041*	
C9	0.2121 (3)	−0.9175 (4)	−0.37668 (11)	0.0352 (6)	
H9A	0.2542	−1.0350	−0.3663	0.042*	
H9B	0.0824	−0.9016	−0.3814	0.042*	
C10	0.2976 (3)	−0.8574 (4)	−0.43586 (12)	0.0381 (6)	
H10A	0.4271	−0.8701	−0.4313	0.046*	
H10B	0.2521	−0.7409	−0.4475	0.046*	
C11	0.2622 (4)	−0.9474 (4)	−0.48561 (13)	0.0474 (7)	
H11A	0.3074	−1.0629	−0.4745	0.071*	
H11B	0.3221	−0.9055	−0.5230	0.071*	
H11C	0.1343	−0.9319	−0.4914	0.071*	
C12	0.3175 (3)	0.9341 (3)	0.23705 (11)	0.0300 (5)	
C13	0.2420 (3)	0.9052 (3)	0.29435 (11)	0.0300 (5)	
H13	0.1816	0.8155	0.3053	0.036*	
C14	0.2538 (3)	1.0058 (3)	0.33557 (11)	0.0301 (5)	
H14	0.2019	0.9852	0.3747	0.036*	
C15	0.3423 (3)	1.1382 (3)	0.31945 (11)	0.0305 (5)	
C16	0.4185 (3)	1.1671 (3)	0.26282 (11)	0.0325 (6)	
H16	0.4781	1.2572	0.2518	0.039*	
C17	0.4079 (3)	1.0642 (3)	0.22178 (11)	0.0320 (6)	
H17	0.4629	1.0829	0.1830	0.038*	
C18	0.3088 (3)	0.8303 (3)	0.19068 (11)	0.0323 (6)	
C19	0.4228 (3)	1.3756 (3)	0.34655 (12)	0.0375 (6)	
H19A	0.3595	1.4478	0.3129	0.045*	
H19B	0.5485	1.3469	0.3337	0.045*	
C20	0.4099 (3)	1.4595 (4)	0.40073 (12)	0.0376 (6)	
H20A	0.4719	1.5539	0.3912	0.045*	
H20B	0.4720	1.3845	0.4340	0.045*	
C21	0.2211 (3)	1.5162 (4)	0.42172 (12)	0.0372 (6)	
H21A	0.1552	1.5829	0.3875	0.045*	
H21B	0.1624	1.4210	0.4354	0.045*	
C22	0.2123 (4)	1.6142 (4)	0.47241 (13)	0.0452 (7)	
H22A	0.2700	1.5464	0.5075	0.068*	
H22B	0.0881	1.6524	0.4830	0.068*	
H22C	0.2731	1.7070	0.4596	0.068*	
C23	0.2365 (4)	−0.0184 (4)	−0.04412 (13)	0.0472 (7)	
H23	0.2711	−0.1324	−0.0368	0.057*	
C24	0.2571 (4)	0.0681 (4)	0.00093 (12)	0.0421 (7)	
H24	0.3000	0.0144	0.0388	0.051*	
C25	0.2135 (3)	0.2354 (3)	−0.01037 (11)	0.0307 (5)	
C26	0.1489 (3)	0.3070 (3)	−0.06606 (11)	0.0332 (6)	
H26	0.1191	0.4211	−0.0756	0.040*	
C27	0.1286 (3)	0.2094 (3)	−0.10754 (12)	0.0347 (6)	
H27	0.0819	0.2593	−0.1453	0.042*	
C28	0.2950 (3)	0.5728 (4)	0.06786 (11)	0.0365 (6)	
H28	0.3359	0.6735	0.0581	0.044*	
C29	0.2834 (3)	0.4853 (3)	0.02218 (11)	0.0329 (6)	
H29	0.3116	0.5271	−0.0182	0.040*	
C30	0.2296 (3)	0.3348 (3)	0.03651 (11)	0.0298 (5)	
C31	0.1889 (3)	0.2808 (3)	0.09596 (11)	0.0334 (6)	
H31	0.1551	0.1775	0.1075	0.040*	
C32	0.1978 (3)	0.3784 (3)	0.13857 (11)	0.0332 (6)	
H32	0.1648	0.3420	0.1790	0.040*	
H1	0.160 (4)	−0.054 (4)	−0.1475 (15)	0.060 (9)*	
H4	0.242 (5)	0.642 (5)	0.1754 (16)	0.065 (10)*	

### (III) 4-n-Butoxybenzoic acid–4,4'-bipyridyl (2/1) Atomic displacement parameters (Å^2^)

**Table:**

	*U*^11^	*U*^22^	*U*^33^	*U*^12^	*U*^13^	*U*^23^
O1	0.0412 (10)	0.0304 (11)	0.0354 (10)	−0.0006 (8)	−0.0033 (8)	−0.0091 (8)
O2	0.0485 (11)	0.0369 (12)	0.0331 (10)	0.0009 (9)	−0.0080 (8)	−0.0085 (8)
O3	0.0326 (9)	0.0333 (11)	0.0377 (10)	0.0069 (8)	−0.0063 (7)	−0.0156 (8)
O4	0.0436 (10)	0.0351 (12)	0.0371 (10)	−0.0067 (8)	−0.0014 (8)	−0.0110 (9)
O5	0.0581 (12)	0.0399 (13)	0.0319 (10)	−0.0076 (10)	0.0004 (9)	−0.0064 (9)
O6	0.0371 (9)	0.0322 (11)	0.0333 (9)	−0.0080 (8)	0.0011 (7)	−0.0089 (8)
N1	0.0455 (12)	0.0327 (14)	0.0337 (12)	−0.0007 (10)	−0.0019 (9)	−0.0092 (10)
N2	0.0328 (10)	0.0357 (14)	0.0340 (11)	0.0000 (9)	−0.0018 (9)	−0.0097 (10)
C1	0.0261 (11)	0.0307 (15)	0.0292 (12)	−0.0020 (10)	0.0019 (9)	−0.0074 (10)
C2	0.0290 (11)	0.0315 (15)	0.0338 (13)	0.0009 (10)	−0.0018 (10)	−0.0079 (11)
C3	0.0280 (11)	0.0358 (16)	0.0321 (13)	0.0023 (11)	−0.0042 (10)	−0.0071 (11)
C4	0.0250 (11)	0.0340 (15)	0.0327 (13)	0.0001 (10)	−0.0016 (9)	−0.0100 (11)
C5	0.0329 (12)	0.0283 (15)	0.0357 (13)	0.0050 (10)	−0.0041 (10)	−0.0088 (11)
C6	0.0293 (11)	0.0369 (16)	0.0304 (12)	−0.0021 (11)	−0.0018 (10)	−0.0078 (11)
C7	0.0324 (12)	0.0317 (15)	0.0328 (13)	−0.0040 (11)	0.0008 (10)	−0.0074 (11)
C8	0.0357 (12)	0.0288 (15)	0.0360 (14)	0.0005 (11)	0.0002 (10)	−0.0088 (11)
C9	0.0351 (12)	0.0359 (16)	0.0360 (13)	−0.0052 (11)	0.0022 (10)	−0.0115 (12)
C10	0.0383 (13)	0.0402 (17)	0.0365 (14)	−0.0050 (12)	0.0019 (11)	−0.0111 (12)
C11	0.0518 (16)	0.054 (2)	0.0385 (15)	−0.0066 (15)	0.0001 (13)	−0.0161 (14)
C12	0.0268 (11)	0.0271 (14)	0.0351 (13)	0.0014 (10)	−0.0069 (10)	−0.0060 (11)
C13	0.0261 (11)	0.0291 (14)	0.0331 (13)	0.0000 (10)	−0.0030 (10)	−0.0043 (11)
C14	0.0280 (11)	0.0326 (15)	0.0296 (12)	−0.0038 (10)	−0.0021 (10)	−0.0053 (11)
C15	0.0265 (11)	0.0289 (15)	0.0353 (13)	−0.0004 (10)	−0.0035 (10)	−0.0058 (11)
C16	0.0312 (12)	0.0315 (15)	0.0338 (13)	−0.0040 (11)	−0.0019 (10)	−0.0035 (11)
C17	0.0290 (11)	0.0341 (15)	0.0309 (13)	−0.0009 (10)	−0.0023 (10)	−0.0037 (11)
C18	0.0333 (12)	0.0297 (15)	0.0317 (13)	0.0019 (11)	−0.0065 (10)	−0.0035 (11)
C19	0.0354 (13)	0.0357 (17)	0.0442 (15)	−0.0095 (12)	0.0020 (11)	−0.0120 (12)
C20	0.0351 (13)	0.0387 (17)	0.0419 (15)	−0.0062 (12)	−0.0037 (11)	−0.0135 (12)
C21	0.0377 (13)	0.0335 (16)	0.0397 (14)	0.0009 (11)	−0.0032 (11)	−0.0101 (12)
C22	0.0491 (16)	0.0396 (18)	0.0474 (16)	−0.0021 (13)	0.0005 (13)	−0.0149 (14)
C23	0.071 (2)	0.0278 (16)	0.0404 (15)	0.0043 (14)	−0.0095 (14)	−0.0083 (13)
C24	0.0577 (17)	0.0306 (16)	0.0344 (14)	0.0049 (13)	−0.0063 (12)	−0.0055 (12)
C25	0.0292 (11)	0.0310 (15)	0.0314 (13)	−0.0004 (10)	0.0003 (10)	−0.0086 (11)
C26	0.0368 (13)	0.0280 (15)	0.0337 (13)	0.0002 (11)	−0.0044 (10)	−0.0061 (11)
C27	0.0351 (13)	0.0339 (16)	0.0340 (13)	0.0014 (11)	−0.0027 (10)	−0.0087 (11)
C28	0.0382 (13)	0.0349 (16)	0.0377 (14)	−0.0058 (12)	−0.0014 (11)	−0.0093 (12)
C29	0.0327 (12)	0.0326 (15)	0.0327 (13)	−0.0018 (11)	0.0012 (10)	−0.0071 (11)
C30	0.0273 (11)	0.0295 (14)	0.0314 (12)	0.0026 (10)	−0.0020 (9)	−0.0086 (11)
C31	0.0346 (12)	0.0316 (15)	0.0326 (13)	−0.0004 (11)	−0.0004 (10)	−0.0064 (11)
C32	0.0323 (12)	0.0324 (15)	0.0312 (13)	0.0036 (11)	0.0003 (10)	−0.0042 (11)

### (III) 4-n-Butoxybenzoic acid–4,4'-bipyridyl (2/1) Geometric parameters (Å, º)

**Table:**

O1—C7	1.319 (3)	C12—C18	1.489 (3)
O1—H1	1.04 (3)	C13—C14	1.382 (3)
O2—C7	1.225 (3)	C13—H13	0.9500
O3—C4	1.363 (3)	C14—C15	1.399 (3)
O3—C8	1.435 (3)	C14—H14	0.9500
O4—C18	1.322 (3)	C15—C16	1.376 (3)
O4—H4	1.00 (4)	C16—C17	1.392 (3)
O5—C18	1.214 (3)	C16—H16	0.9500
O6—C15	1.362 (3)	C17—H17	0.9500
O6—C19	1.438 (3)	C19—C20	1.506 (3)
N1—C27	1.325 (4)	C19—H19A	0.9900
N1—C23	1.335 (4)	C19—H19B	0.9900
N2—C28	1.334 (3)	C20—C21	1.518 (4)
N2—C32	1.337 (3)	C20—H20A	0.9900
C1—C6	1.381 (4)	C20—H20B	0.9900
C1—C2	1.398 (3)	C21—C22	1.516 (4)
C1—C7	1.475 (3)	C21—H21A	0.9900
C2—C3	1.381 (3)	C21—H21B	0.9900
C2—H2	0.9500	C22—H22A	0.9800
C3—C4	1.390 (4)	C22—H22B	0.9800
C3—H3	0.9500	C22—H22C	0.9800
C4—C5	1.389 (3)	C23—C24	1.382 (4)
C5—C6	1.387 (3)	C23—H23	0.9500
C5—H5	0.9500	C24—C25	1.389 (4)
C6—H6	0.9500	C24—H24	0.9500
C8—C9	1.505 (3)	C25—C26	1.386 (3)
C8—H8A	0.9900	C25—C30	1.483 (3)
C8—H8B	0.9900	C26—C27	1.383 (3)
C9—C10	1.504 (4)	C26—H26	0.9500
C9—H9A	0.9900	C27—H27	0.9500
C9—H9B	0.9900	C28—C29	1.384 (3)
C10—C11	1.523 (3)	C28—H28	0.9500
C10—H10A	0.9900	C29—C30	1.394 (4)
C10—H10B	0.9900	C29—H29	0.9500
C11—H11A	0.9800	C30—C31	1.382 (3)
C11—H11B	0.9800	C31—C32	1.383 (3)
C11—H11C	0.9800	C31—H31	0.9500
C12—C13	1.387 (3)	C32—H32	0.9500
C12—C17	1.390 (3)		
			
C7—O1—H1	107 (2)	C15—C16—C17	119.9 (2)
C4—O3—C8	117.16 (19)	C15—C16—H16	120.1
C18—O4—H4	105 (2)	C17—C16—H16	120.1
C15—O6—C19	117.34 (19)	C12—C17—C16	120.5 (2)
C27—N1—C23	117.5 (2)	C12—C17—H17	119.7
C28—N2—C32	118.2 (2)	C16—C17—H17	119.7
C6—C1—C2	118.9 (2)	O5—C18—O4	123.1 (2)
C6—C1—C7	118.8 (2)	O5—C18—C12	123.0 (2)
C2—C1—C7	122.3 (2)	O4—C18—C12	113.9 (2)
C3—C2—C1	120.5 (2)	O6—C19—C20	108.0 (2)
C3—C2—H2	119.7	O6—C19—H19A	110.1
C1—C2—H2	119.7	C20—C19—H19A	110.1
C2—C3—C4	120.0 (2)	O6—C19—H19B	110.1
C2—C3—H3	120.0	C20—C19—H19B	110.1
C4—C3—H3	120.0	H19A—C19—H19B	108.4
O3—C4—C5	123.5 (2)	C19—C20—C21	113.9 (2)
O3—C4—C3	116.6 (2)	C19—C20—H20A	108.8
C5—C4—C3	119.9 (2)	C21—C20—H20A	108.8
C6—C5—C4	119.6 (2)	C19—C20—H20B	108.8
C6—C5—H5	120.2	C21—C20—H20B	108.8
C4—C5—H5	120.2	H20A—C20—H20B	107.7
C1—C6—C5	121.1 (2)	C22—C21—C20	112.6 (2)
C1—C6—H6	119.5	C22—C21—H21A	109.1
C5—C6—H6	119.5	C20—C21—H21A	109.1
O2—C7—O1	122.9 (2)	C22—C21—H21B	109.1
O2—C7—C1	122.4 (2)	C20—C21—H21B	109.1
O1—C7—C1	114.7 (2)	H21A—C21—H21B	107.8
O3—C8—C9	108.3 (2)	C21—C22—H22A	109.5
O3—C8—H8A	110.0	C21—C22—H22B	109.5
C9—C8—H8A	110.0	H22A—C22—H22B	109.5
O3—C8—H8B	110.0	C21—C22—H22C	109.5
C9—C8—H8B	110.0	H22A—C22—H22C	109.5
H8A—C8—H8B	108.4	H22B—C22—H22C	109.5
C10—C9—C8	114.8 (2)	N1—C23—C24	123.5 (3)
C10—C9—H9A	108.6	N1—C23—H23	118.3
C8—C9—H9A	108.6	C24—C23—H23	118.3
C10—C9—H9B	108.6	C23—C24—C25	118.5 (3)
C8—C9—H9B	108.6	C23—C24—H24	120.7
H9A—C9—H9B	107.5	C25—C24—H24	120.7
C9—C10—C11	112.5 (2)	C26—C25—C24	118.2 (2)
C9—C10—H10A	109.1	C26—C25—C30	120.8 (2)
C11—C10—H10A	109.1	C24—C25—C30	121.0 (2)
C9—C10—H10B	109.1	C27—C26—C25	118.9 (3)
C11—C10—H10B	109.1	C27—C26—H26	120.6
H10A—C10—H10B	107.8	C25—C26—H26	120.6
C10—C11—H11A	109.5	N1—C27—C26	123.4 (2)
C10—C11—H11B	109.5	N1—C27—H27	118.3
H11A—C11—H11B	109.5	C26—C27—H27	118.3
C10—C11—H11C	109.5	N2—C28—C29	122.9 (2)
H11A—C11—H11C	109.5	N2—C28—H28	118.5
H11B—C11—H11C	109.5	C29—C28—H28	118.5
C13—C12—C17	119.2 (2)	C28—C29—C30	118.8 (2)
C13—C12—C18	123.0 (2)	C28—C29—H29	120.6
C17—C12—C18	117.7 (2)	C30—C29—H29	120.6
C14—C13—C12	120.5 (2)	C31—C30—C29	118.0 (2)
C14—C13—H13	119.7	C31—C30—C25	120.5 (2)
C12—C13—H13	119.7	C29—C30—C25	121.5 (2)
C13—C14—C15	119.8 (2)	C30—C31—C32	119.5 (2)
C13—C14—H14	120.1	C30—C31—H31	120.3
C15—C14—H14	120.1	C32—C31—H31	120.3
O6—C15—C16	124.4 (2)	N2—C32—C31	122.5 (2)
O6—C15—C14	115.6 (2)	N2—C32—H32	118.7
C16—C15—C14	120.0 (2)	C31—C32—H32	118.7
			
C6—C1—C2—C3	1.2 (3)	C18—C12—C17—C16	179.0 (2)
C7—C1—C2—C3	−177.4 (2)	C15—C16—C17—C12	1.4 (4)
C1—C2—C3—C4	0.2 (4)	C13—C12—C18—O5	175.6 (2)
C8—O3—C4—C5	4.5 (3)	C17—C12—C18—O5	−5.2 (4)
C8—O3—C4—C3	−176.1 (2)	C13—C12—C18—O4	−5.6 (3)
C2—C3—C4—O3	179.5 (2)	C17—C12—C18—O4	173.5 (2)
C2—C3—C4—C5	−1.1 (4)	C15—O6—C19—C20	179.9 (2)
O3—C4—C5—C6	180.0 (2)	O6—C19—C20—C21	−62.8 (3)
C3—C4—C5—C6	0.5 (4)	C19—C20—C21—C22	−174.4 (2)
C2—C1—C6—C5	−1.8 (3)	C27—N1—C23—C24	2.1 (4)
C7—C1—C6—C5	176.9 (2)	N1—C23—C24—C25	−2.6 (5)
C4—C5—C6—C1	0.9 (4)	C23—C24—C25—C26	1.0 (4)
C6—C1—C7—O2	−4.7 (4)	C23—C24—C25—C30	178.9 (3)
C2—C1—C7—O2	174.0 (2)	C24—C25—C26—C27	0.8 (4)
C6—C1—C7—O1	176.3 (2)	C30—C25—C26—C27	−177.1 (2)
C2—C1—C7—O1	−5.1 (3)	C23—N1—C27—C26	−0.2 (4)
C4—O3—C8—C9	−178.40 (19)	C25—C26—C27—N1	−1.3 (4)
O3—C8—C9—C10	66.9 (3)	C32—N2—C28—C29	1.6 (4)
C8—C9—C10—C11	178.0 (2)	N2—C28—C29—C30	−2.3 (4)
C17—C12—C13—C14	1.0 (4)	C28—C29—C30—C31	0.5 (4)
C18—C12—C13—C14	−179.8 (2)	C28—C29—C30—C25	179.6 (2)
C12—C13—C14—C15	0.1 (4)	C26—C25—C30—C31	140.9 (3)
C19—O6—C15—C16	3.6 (4)	C24—C25—C30—C31	−37.0 (3)
C19—O6—C15—C14	−175.7 (2)	C26—C25—C30—C29	−38.2 (3)
C13—C14—C15—O6	178.8 (2)	C24—C25—C30—C29	144.0 (3)
C13—C14—C15—C16	−0.5 (4)	C29—C30—C31—C32	1.8 (4)
O6—C15—C16—C17	−179.5 (2)	C25—C30—C31—C32	−177.3 (2)
C14—C15—C16—C17	−0.3 (4)	C28—N2—C32—C31	0.9 (4)
C13—C12—C17—C16	−1.8 (4)	C30—C31—C32—N2	−2.6 (4)

### (III) 4-n-Butoxybenzoic acid–4,4'-bipyridyl (2/1) Hydrogen-bond geometry (Å, º)

*Cg*1 and *Cg*2 are the centroids of the C1–C6 and C12–C17 rings, respectively.

**Table:**

*D*—H···*A*	*D*—H	H···*A*	*D*···*A*	*D*—H···*A*
O1—H1···N1	1.04 (3)	1.56 (3)	2.600 (3)	173 (3)
O4—H4···N2	1.00 (4)	1.64 (4)	2.636 (3)	172 (4)
C24—H24···O5^i^	0.95	2.47	3.408 (3)	171
C29—H29···O2^ii^	0.95	2.53	3.456 (3)	164
C2—H2···*Cg*2^iii^	0.95	2.98	3.754 (3)	139
C8—H8*B*···*Cg*2^iv^	0.99	2.68	3.518 (3)	143
C19—H19*B*···*Cg*1^v^	0.99	2.77	3.586 (3)	140

Symmetry codes: (i) *x*, *y*−1, *z*; (ii) *x*, *y*+1, *z*; (iii) −*x*, −*y*+1, −*z*; (iv) −*x*+1, −*y*, −*z*; (v) −*x*+1, −*y*+1, −*z*.
